# Water‐borne and plasma corticosterone are not correlated in spotted salamanders

**DOI:** 10.1002/ece3.5831

**Published:** 2019-11-18

**Authors:** Alice R. Millikin, Sarah K. Woodley, Drew R. Davis, Ignacio T. Moore, James T. Anderson

**Affiliations:** ^1^ School of Natural Resources West Virginia University Morgantown WV USA; ^2^ Department of Biological Sciences Duquesne University Pittsburgh PA USA; ^3^ School of Earth, Environmental, and Marine Sciences University of Texas Rio Grande Valley South Padre Island TX USA; ^4^ Department of Biological Sciences Virginia Tech Blacksburg VA USA

**Keywords:** *Ambystoma maculatum*, glucocorticoid, hormones, noninvasive, validation

## Abstract

Water‐borne hormone measurement is a noninvasive method suitable for amphibians of all sizes that are otherwise difficult to sample. For this method, containment‐water is assayed for hormones released by the animal. Originally developed in fish, the method has expanded to amphibians, but requires additional species‐specific validations. We wanted to determine physiological relevance of water‐borne corticosterone in spotted salamanders (*Ambystoma maculatum*) by comparing concentrations to those taken using established corticosterone sampling methods, such as plasma. Using a mixture of field and laboratory studies, we compared water‐borne corticosterone levels to other traditional methods of sampling corticosterone for spotted salamander larvae, metamorphs, and adults. Despite multiple attempts, and detecting differences between age groups, we found no correlations between water‐borne and plasma corticosterone levels in any age group. Water‐borne sampling measures a rate of release; whereas plasma is the concentration circulating in the blood. The unique units of measurement may inherently prevent correlations between the two. These two methods may also require different interpretations of the data and the physiological meaning. We also note caveats with the method, including how to account for differences in body size and life history stages. Collectively, our results illustrate the importance of careful validation of water‐borne hormone levels in each species in order to understand its physiological significance.

## Graphical abstract

Water‐borne and plasma corticosterone concentrations are not correlated in spotted salamander larvae, metamorphs, or adults. The two sampling methods produce unique units of measurement that might require separate interpretations of the data and physiological relevance.

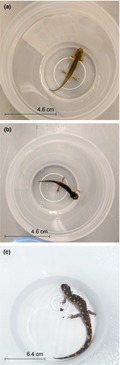

## 1 INTRODUCTION

Conservation physiology provides additional avenues to assess population health by measuring physiological responses to habitat quality (Wikelski & Cooke, 2006). These physiological responses could predict population declines and facilitate detection of vulnerable populations (McCormick & Romero, 2017). Corticosterone is a glucocorticoid hormone that is a useful parameter to measure sublethal impacts and could act as an indicator of habitat quality (Homyack, 2010; McCormick & Romero, 2017; Romero & Wikelski, 2001). When amphibians encounter environmental changes that require a physiological or behavioral response, the hypothalamus–pituitary–interrenal (HPI) axis is activated, releasing corticosterone to make energy available (McEwen & Wingfield, 2003; Romero, 2004). This allows amphibians to maintain allostasis; however, sustained elevated corticosterone concentrations can suppress the immune system and growth (McEwen & Wingfield, 2003).

Corticosterone levels of wild populations are typically interpreted two ways. The first measures baseline corticosterone levels of individuals by sampling within 3 min of capture to obtain corticosterone levels prior to elevation that can occur due to handling (Romero & Reed, 2005). Corticosterone levels can also be quantified by comparing baseline levels to corticosterone levels after adrenocorticotropic hormone (ACTH) challenge or physical agitation. This involves collecting a sample within 3 min of capture and again after the treatment (ACTH challenge or physical agitation). If there is no change after a treatment previously shown to increase corticosterone in the study species, one possible explanation is that baseline levels were already elevated, which downregulated the HPI axis response.

**Figure 1:**
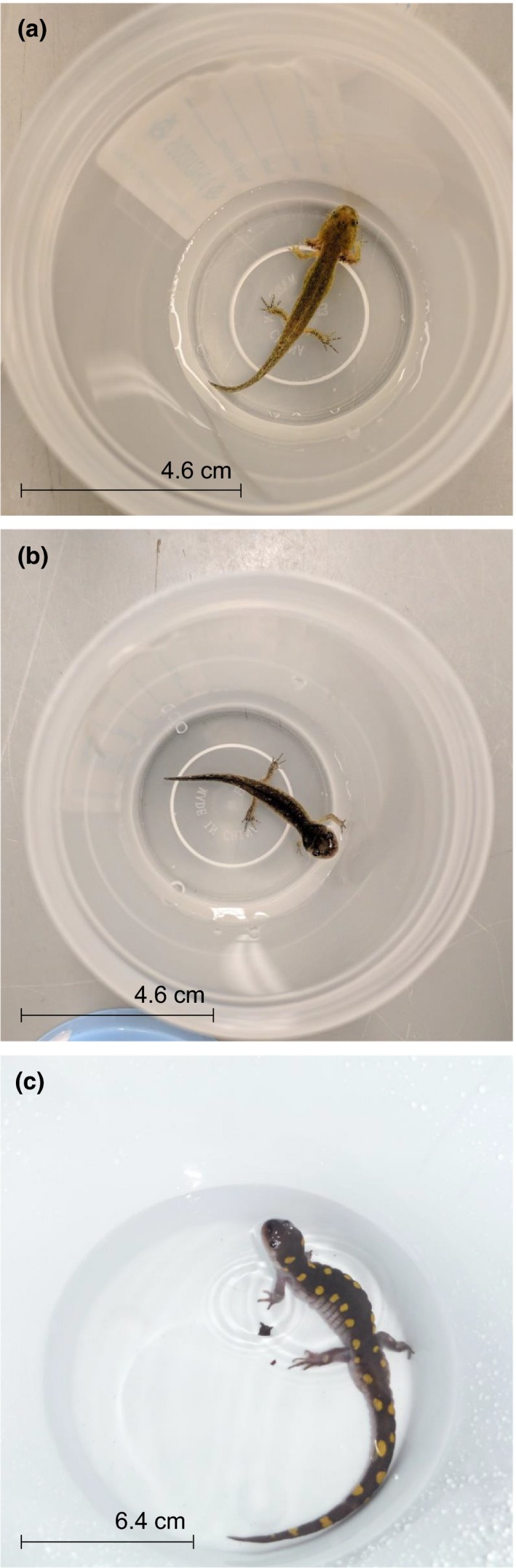
Photographs of a spotted salamander larva (a), metamorph (b), and adult (c) in individual containers of water for water‐borne hormone sampling

Water‐borne hormone sampling is a noninvasive method originally used in fish (Félix, Faustino, Cabral, & Oliveira, 2013; Scott & Ellis, 2007), which has recently expanded to amphibians (Figure 1; Baugh, Bastien, Still, & Stowell, 2018; Gabor et al., 2013, 2016). While contained in water, the animal releases corticosterone through the gills, skin, and urine that can later be extracted (Félix et al., 2013; Gabor et al., 2013; Scott & Ellis, 2007). Water‐borne corticosterone is interpreted as a cumulative measure of the hormone over a set period of time, usually 1 hr (Gabor et al., 2013; Scott & Ellis, 2007). This reflects the corticosterone release rate, or the rate of secretion of the hormone (Dantzer, Fletcher, Boonstra, & Sheriff, 2014; Gabor et al., 2013; Scott & Ellis, 2007).

Documented correlations between water‐borne corticosterone and treatments linked to adverse physiological effects indicate biological significance for water‐borne hormone sampling (Charbonnier et al., 2018; Gabor, Fisher, & Bosch, 2015; Holmes et al., 2016). Spotted salamander larvae in high intraspecific densities and juveniles in low‐moisture environments had elevated water‐borne corticosterone levels and the larvae had reduced growth (Charbonnier et al., 2018). More aggressive and more severe infections of *Batrachochytrium dendrobatidis* (*Bd*) were associated with higher water‐borne corticosterone levels in the Mallorcan midwife toad (*Alytes muletensis*) and common midwife toad (*A. obstetricians*; Gabor et al., 2015). Higher water‐borne corticosterone levels were associated with higher ranavirus infection intensity in larval western tiger salamanders (*Ambystoma mavortium*; Davis, Ferguson, Schwarz, & Kerby, 2019). In common midwife toads, *Bd* infection and higher corticosterone levels were associated with decreased ability of their righting reflex (Gabor et al., 2015). African clawed frogs (*Xenopus laevis*) in tanks with an unnatural background color (white) had higher levels of water‐borne corticosterone along with greater body mass loss (Holmes et al., 2016). The measurable responses in water‐borne corticosterone levels coupled with biological responses indicate an association between water‐borne corticosterone levels and the treatments that is biologically relevant.

Water‐borne sampling can be conducted on amphibians of any size, which makes it useful for species that are difficult to sample like larval spotted salamanders (*Ambystoma maculatum*; Figure 1). Larval spotted salamanders, which can weigh <1 g and measure less than 4 cm total length, are often too small to obtain detectable whole‐body corticosterone levels or sufficient blood samples, even when the animal is sacrificed (A.R. Millikin, personal observation, this paper). This species reproduces and completes metamorphosis in vernal pools, small wetlands vulnerable to destruction (Calhoun, Arrigoni, Brooks, Hunter, & Richter, 2014). Water‐borne hormone sampling could provide a beneficial tool to monitor the larval physiological health in sensitive wetlands.

Interpreting water‐borne hormone data requires multiple levels of validation. Chemical validation and quality controls determine if the assays are reporting reliable values for corticosterone (Behringer & Deschner, 2017). Biological validation determines if changes in hormone levels are biologically relevant by demonstrating associations with either changes in the environment or the resulting effects to the animal like reduced growth. Finally, physiological validation demonstrates a link between water‐borne corticosterone levels and other measures of corticosterone in the same animal (Scott & Ellis, 2007). The physiological validation is necessary to create a strong foundation for appropriate applications of the method (Madliger, Love, Hultine, & Cooke, 2018). Most research considers water‐borne hormone sampling valid for a species if it is correlated with hormone levels in plasma (Gabor et al., 2013; Scott & Ellis, 2007). Positive correlations between water‐borne corticosterone and plasma corticosterone levels have been found in four amphibian species including adult Túngara frogs (*Physalaemus pustulosus*; *r* = .46; Baugh et al., 2018), larval western tiger salamanders (*r^2^* = .60; Davis et al., 2019), adult San Marcos salamanders (*Eurycea nana*; *r* = .87), and adult common midwife toads (*r* = .86; Gabor et al., 2013). To demonstrate detection of physiologically relevant changes in corticosterone, studies often attempt to increase corticosterone in some individuals by injecting them with ACTH or by exposing them to physical agitation (Baugh et al., 2018; Gabor, Davis, Kim, Zabierek, & Bendik, 2018; Gabor et al., 2016).

We wanted to determine whether water‐borne corticosterone was correlated with plasma corticosterone levels in spotted salamanders in order to physiologically validate the method for this species. Because our main goal was to determine whether natural individual variation in corticosterone could be detected using water‐borne sampling methods, we started by assessing the correlation between water‐borne and whole‐body corticosterone of larvae in the field. Next, we collected larvae and reared them in the laboratory until they were large enough to collect plasma. We compared water‐borne and plasma corticosterone in these laboratory‐reared larvae and metamorphs. Half of the laboratory‐reared animals were randomly selected for physical agitation to induce an increase in corticosterone to compare with animals under baseline conditions. If we detected an increase in corticosterone in response to agitation in both water‐borne and plasma samples, it would indicate physiological relevance for water‐borne corticosterone in this species (Behringer & Deschner, 2017). Finally, to broaden the applicability of the sampling method, we evaluated whether there was a correlation between water‐borne and plasma corticosterone levels of adult spotted salamanders in the field. We tested for differences in corticosterone levels among larvae, metamorphs, and adults to test whether water‐borne corticosterone would follow the same trend as plasma and to determine if the physiological factor, age, impacted corticosterone levels (Behringer & Deschner, 2017).

## 2 MATERIALS AND METHODS

This study was completed with approval from West Virginia University Institutional Animal Care and Use Committee (15‐0409.3), Duquesne University Institutional Animal Care and Use Committee (1609‐09), the U.S. Forest Service, West Virginia Division of Natural Resources (Scientific Collecting Permit 2015.133, 2016.205, 2017.073), and the Pennsylvania Fish and Boat Commission (Scientific Collecting Permit 2017‐01‐0048).

### 2.1 Larvae and metamorphs

#### 2.1.1 Water‐borne versus whole‐body corticosterone

We used a seine and dip net to collect spotted salamander larvae in created vernal pools on Cheat Mountain in Monongahela National Forest, WV. On 8 June 2015 at 1429 hr, we collected 16 larvae with an average weight of 0.047 ± 0.005 g (range: 0.019–0.076 g). Each larva was placed in an individual high‐density polyethylene specimen cup (Dynarex Model 4254) in 20 ml of distilled water (premixed with Kent R/O Right Water Conditioner to prevent osmotic shock) and removed after 1 hr. Upon removal from the water‐borne sample, larvae were immediately placed in individual microcentrifuge tubes and put in a dry ice and ethanol bath. We repeated the experiment, collecting 30 larger larvae (average weight 0.393 ± 0.027 g, ranging from 0.100 to 0.710 g; stages 17–18; Watson & Russell, 2000) on 13 June 2016 at 1232 hr. We pooled samples (3 larvae/sample) for whole‐body testing to increase likelihood that corticosterone levels would be detectable. We pooled the same individuals' water‐borne samples for comparison. Samples were stored at −20°C until analysis.

#### 2.1.2 Water‐borne versus plasma corticosterone

Next, we tested the relationship between water‐borne corticosterone and plasma corticosterone both at baseline and after stimulating the animals in an effort to increase corticosterone. To do so, we collected 29 larval spotted salamanders on 8 July 2017 at 1418 hr from created vernal pools on Cheat Mountain in Monongahela National Forest, WV using dip nets and seines. Larvae were reared in the laboratory at West Virginia University for 19 days in 3 L of water at a density of 1 larvae/L, resulting in 3 larvae per container. Container water was initially from the source wetland, then animals were transitioned to dechlorinated tap water. Larvae were housed at 22°C with a photoperiod of 14 hr light: 10 hr dark and fed thawed blood worms ad libitum. We changed water daily to prevent accumulation of debris, feces, food, and algae. During the 19 days in the laboratory, 13 of the 29 larvae completed metamorphosis. After metamorphosis, salamanders were provided both wet and dry refuge along with mesh cover. At the time of the experiment, the 16 larvae were in developmental stages 18–21, had a total length (TL) of 4.53 ± 0.09 cm, a snout–vent length (SVL) of 2.83 ± 0.07 cm, and mass of 0.68 ± 0.04 g (Watson & Russell, 2000). The other 13 were metamorphs at stage 22, had a TL of 5.02 ± 0.10 cm, SVL of 3.06 ± 0.06 cm, and mass of 0.71 ± 0.04 g (Watson & Russell, 2000).

Larvae and metamorphs were sampled for water‐borne corticosterone as described above. All water‐borne hormone sampling of laboratory‐reared salamanders occurred between 1200 and 1700 hr. To determine if we could detect a change in water‐borne hormones in response to physical agitation, half of the laboratory‐reared larvae and metamorphs were randomly assigned to one of two treatment groups. The baseline group included eight larvae and six metamorphs. The physical agitation group (hereafter agitation) included eight larvae and seven metamorphs. For the agitation group, we physically agitated salamanders while in their individual water‐borne sampling cups by chasing them with blunt tweezers until they no longer exhibited a righting response ($\overset{-}{X}$ = 30 min 54 s, range: 20–50 min). For both the baseline and agitation groups, immediately after collecting a water‐borne hormone sample, salamanders were anesthetized in MS‐222, decapitated, and a blood sample was collected within 3 min of removal from the water to test plasma levels of corticosterone (Romero & Reed, 2005). Within 4 hr of collection, blood samples were centrifuged for 5 min and plasma was collected ($\overset{-}{X}$ = 6.34 µl, range: 1.50–13.50 µl) and stored at −80°C until analysis. Water‐borne samples were stored at −20°C until analysis.

#### 2.1.3 Corticosterone extraction and assays

Whole‐body corticosterone from field‐caught larvae was measured with radioimmunoassay (RIA) at Virginia Tech following methods described in Belden, Moore, Mason, Wingfield, and Blaustein (2003) with the following modifications. Each larva was weighed, resuspended in 4.5 ml of distilled water, ground with a tissue tearer for a minimum of 15 s, and transferred to a new vial for extraction. The original vial was rinsed with 0.5 ml of distilled water and added to the extraction vial. Each sample was then triple extracted with 3 ml of dicholoromethane and centrifuged at 2,000 rpm. These extracts were dried and then resuspended in phosphate‐buffered saline with gelatin before beginning the assay. Samples were incubated overnight with 100 µl of antibody (#B3‐163, Esoterix Endocrinology) and 100 µl of tritiated steroid. Unbound steroid was removed using dextran‐coated charcoal, and bound steroid was collected in scintillation vials. All samples were assayed in duplicate.

Both water samples and plasma samples from laboratory‐reared larvae and metamorphs were sent to the Endocrine Technologies Core (ETC) at the Oregon National Primate Research Center (ONPRC), where corticosterone was extracted and then measured by RIA. Plasma samples were combined with 0.1% gel‐PBS, and steroids were extracted with 5 ml diethyl ether by vigorous inversion for 3 min. Samples were centrifuged for 5 min (2000 *g*, 4°C), and aqueous phase was frozen using a dry ice bath. The organic phase was decanted into a new 13 × 100 mm tube and dried under forced air in a 37°C water bath. Samples were then redissolved in 0.1% gel‐PBS and assayed for corticosterone using an in‐house RIA. A standard curve ranging from 5 pg/tube to 1,000 pg/tube was created using 3H‐corticosterone (American Radiolabeled Chemicals). The antibody used was a commercially available anticorticosterone antibody (Abcam). Hormonal values were corrected for extraction losses determined by radioactive trace recovery at the same time as sample extraction, which ranged between 90% and 92%. The sensitivity was 5 pg/tube. Recovery for the plasma assay was 92.6%, and intra‐assay variation was 7.3%. For water samples, corticosterone was extracted using Strata C18‐E (55 µm, 70 Å), 200 mg/3 ml solid‐phase extraction (SPE) columns (Phenomenex). Columns were conditioned using methanol and equilibrated with water prior to addition of 5 ml of water sample (2.5 ml × 2). Columns were washed with 40% methanol and steroids eluted into a new 13 × 100 mm tube using 90% methanol. Samples were then dried down under forced air in a 37°C water bath and assayed as described above. Recovery for the water assay was 80.39% and intra‐assay variation was 12.4%.

### 2.2 Adult salamanders

#### 2.2.1 Water‐borne versus plasma corticosterone

In Allegheny County, PA, we hand caught 28 adult spotted salamanders that were located in and migrating to ephemeral breeding pools on 6 March 2017 and 7 March 2017. Sampling occurred after dark between 1957 and 0058 hr in rainy weather with temperatures between 5 and 12°C. We immediately placed salamanders in individual high‐density polyethylene buckets filled with 550 ml of distilled water (premixed with Kent R/O Right Water Conditioner) to collect water‐borne hormones. After 1 hr, salamanders were removed from the buckets and a blood sample was collected. If salamanders were contained in the buckets for longer than 1 hr before collecting a blood sample (*n* = 26 out of 28 samples, $\overset{-}{X}$ = 1 hr 17 min, max = 1 hr 51 min), then final water‐borne corticosterone levels were divided by number of hours contained (Gabor et al., 2013). We collected blood samples from adults from the caudal tail vein within 3 min of removal from buckets. Using a 21‐gauge needle to access the vein, we collected blood with heparinized hematocrit capillary tubes (Woodley & Porter, 2016). After collecting water and plasma samples, we measured, weighed, and released all adult spotted salamanders. Adult salamanders sampled averaged 17.44 ± 0.25 cm TL, 9.16 ± 0.12 cm SVL, and 16.16 ± 0.35 g. Within 6 hr of collection, blood samples were centrifuged for 5 min and plasma was collected. Water‐borne and plasma samples were stored at −20°C for future analysis.

#### 2.2.2 Water‐borne hormone extraction

Adult salamander water‐borne hormone samples were assayed at Duquesne University with Corticosterone ELISA kits (#501320, Cayman Chemicals, Inc.). Water‐borne hormone samples were filtered with Q8 Whatman filter paper to remove suspended particles. We primed C18 solid‐phase extraction (SPE) columns (SepPak Vac 3 cc/200 mg; Waters, Inc.) using 4 ml of HPLC‐grade methanol and 4 ml of nanopure water. Water‐borne corticosterone samples were pulled into Tygon tubing (Saint‐Gobain formulation 2475) and through the SPE column. Parafilm wax provided a seal between the tubing and SPE column opening. Corticosterone was eluted off the SPE column into glass test tubes using 4 ml methanol. Test tubes were placed in a 42°C water bath and methanol was evaporated with nitrogen gas using an Evap‐o‐rac. Samples were checked every 15 min until all methanol was evaporated (about 1 hr). Corticosterone left in the test tubes was resuspended in 400 μl of 95% ELISA buffer/5% ethanol then diluted 1:10 due to high concentrations of corticosterone. Each sample was vortexed for 10 s, covered, and refrigerated overnight or up to two nights. If they could not be assayed within a couple days, samples were frozen at −20°C.

#### 2.2.3 Water‐borne hormone measurement using ELISA

Samples and kit reagents were brought to room temperature and vortexed before plating. Samples were plated in duplicate using ELISA kits and a plate reader set to 415 nm (Bio‐Rad 3550). Each ELISA plate included two positive and two negative controls in duplicate. The negative controls were distilled water with R/O Right Water Conditioner that had not held salamanders. Matrix effects (Scott & Ellis, 2007) result in negative controls presenting with detectable levels of corticosterone. It is standard to subtract the concentrations detected in negative controls from the sample values. The average background corticosterone recorded in our negative control samples was subtracted from salamander water‐borne hormone samples. For positive controls, we combined water‐borne corticosterone samples from an additional 30 larvae into one sample. The total volume was then aliquoted into individual vials of 20 ml to include as a positive control on each plate to determine coefficients of variation. For each plate, two positive and two negative controls (20 ml of each) were run through C18 columns, extracted and eluted at the same time as water‐borne corticosterone samples. Two positive control samples were run in duplicate on every plate to assess inter‐ and intra‐assay variation. Intra‐assay variation was 18.3% and 20.2%, the interassay variation was 29.0%. This is within range for water‐borne hormone assays: max intra‐assay variation of 23.1% (Gabor et al., 2013) and max interassay variation of 35.3% (Gabor et al., 2016). Additionally, we expect our variation to be higher because it incorporated variation starting from the step of running the sample through C18 columns. Other studies often pool samples for a positive control after the final step of resuspension, therefore, not incorporating variation from the extraction process.

To validate ELISA kits, we used a subset of water‐borne negative control samples and some extra water‐borne samples from free‐living salamanders to compare values from ELISAs at Duquesne University to those measured with RIAs at the ETC at ONPRC. In both ELISA and RIA, negative control samples had less corticosterone than samples from salamanders. In addition, salamander water‐borne hormone values from ELISA and RIA were correlated (Pearson correlation: *n* = 20, *r* = .74, *p* = .0002).

Finally, chemical validation of the ELISA assay was demonstrated with quantitative recovery and parallelism, which test accurate measurement of corticosterone within the assay itself. For recovery, a positive control sample was diluted 1:2 and combined in equal parts with the eight standards of the ELISA standard curve. The diluted positive control and the control + standard samples were plated in duplicate. Expected concentration was measured as ([known corticosterone concentration in the unaltered pooled sample + concentration of the standard]/2). Recovery was calculated by observed concentration divided by expected concentration. Minimum observed recovery was 64% (Millikin, Woodley, Davis, & Anderson, 2019). Observed and expected values were linearly related (slope = 1.40, *F*_(1,7)_ = 3,945, *R* ^2^ = .998, *p* < .0001; Millikin et al., 2019). We demonstrated parallelism between the standard curve of the assay and successive dilutions of the positive control samples (based on eight dilutions 1:1–1:128, each run in duplicate) by showing the curves were not significantly different (*t*_(12)_ = 1.26, *p* = .23; Millikin et al., 2019).

#### 2.2.4 Plasma samples

Plasma samples were sent to the ETC at ONPRC, where they were extracted and then assayed for corticosterone using RIA following the methods described earlier. Plasma assay recovery was 85.8% and intra‐assay variation was 10.6%.

### 2.3 Analysis

We present water‐borne corticosterone in units of pg/snout–vent length/hour (pg/SVL/hr) to attempt to control for body size and provide data comparable to other published studies (Gabor et al., 2013). We also compare to water‐borne corticosterone in units of pg/body weight/hour (pg/g/hr) since some research has controlled for body size using mass (Charbonnier et al., 2018). Due to missing mass data from one adult female salamander, only 27 of the 28 adults could be measured for water‐borne corticosterone by weight (pg/g/hr).

All analyses were conducted in R (R Core Team, 2017). We used Spearman's rank correlation to test for correlation between the water‐borne corticosterone and plasma corticosterone of laboratory‐reared larvae, metamorphs, and field‐caught adults (*α* = .05). Because we were looking for a correlation between plasma and water‐borne corticosterone, we included corticosterone levels of both baseline and agitation groups in analysis. Six of 28 adult salamanders sampled took 4–5 min to collect sufficient blood samples, failing to meet the <3 min criteria. Because these data did not alter the trend, they were included in analysis to increase sample size and power of detection.

We tested normality for laboratory‐reared salamanders with a Shapiro–Wilk test. We natural log‐transformed larval water‐borne and plasma data to meet assumptions of normality for *t* tests to compare corticosterone levels between baseline and agitation levels (*α* = .05). Due to excessive zeros in metamorphs, we used Kruskal–Wallis to compare corticosterone levels between baseline and agitation groups. We also compared corticosterone levels of baseline and agitation groups with larval and metamorph data combined using Kruskal–Wallis. Corticosterone levels were compared across age groups (adult, larvae, and metamorphs) using Kruskal–Wallis followed by Dunn test (*α* = .05; package “dunn.test”, Dinno, 2017).

## 3 RESULTS

**Figure 2:**
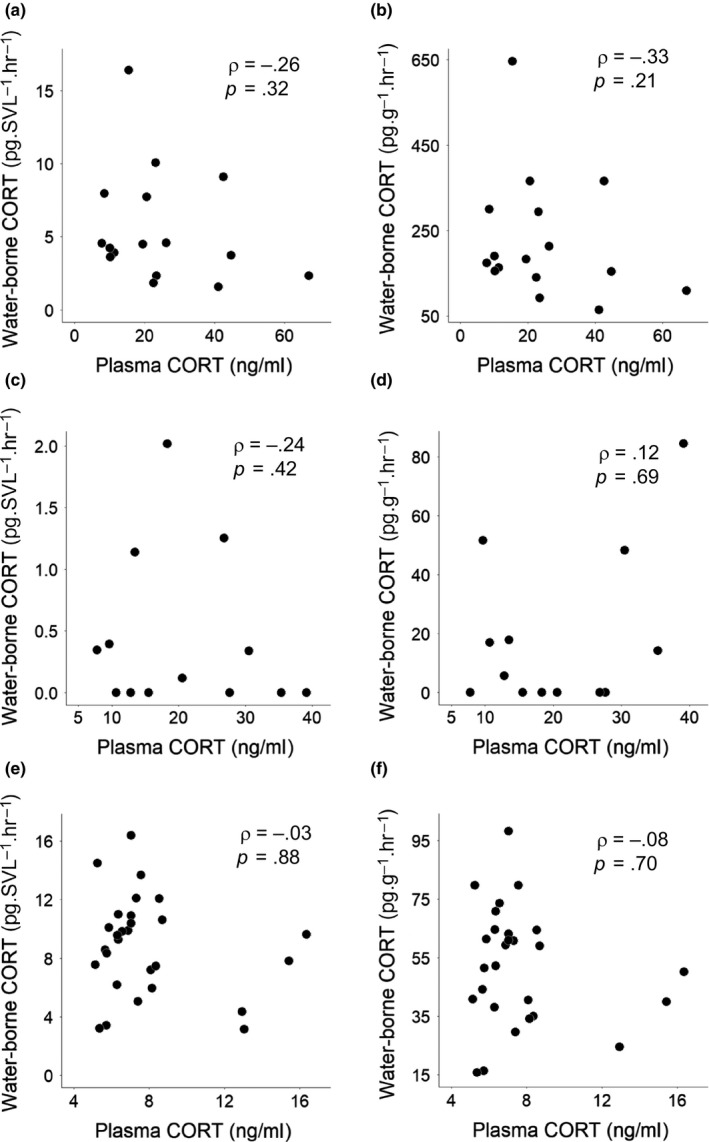
Biplots of spotted salamander plasma corticosterone (CORT) and water‐borne CORT lacking correlations. Left side: corticosterone is presented in units of pg/SVL/hr. Right side: corticosterone is presented in units of pg/g/hr. From top to bottom: larvae (a, b), metamorphs (c, d), and adults (e, f). Spearman's *rho* and *p* values are presented on each graph

**Figure 3:**
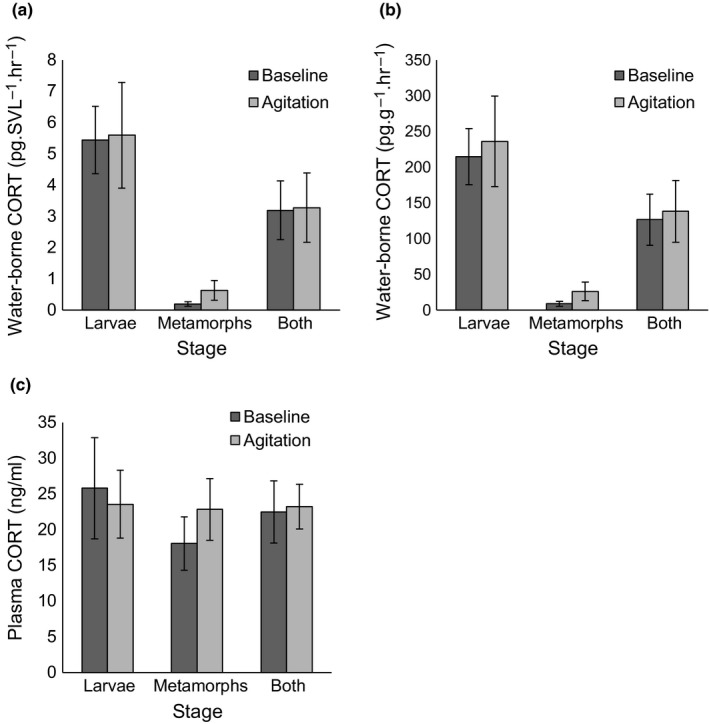
Graphs displaying untransformed corticosterone (CORT) ± *SE* for spotted salamander baseline and agitation groups measured in different subsets of larvae (*n* = 8, 8), metamorphs (*n* = 6, 7), and both stages (*n* = 14, 15) combined for water‐borne CORT (a, b) and plasma levels (c)

Individuals tested for whole‐body corticosterone in 2015 and pooled larvae whole‐body samples in 2016 both had corticosterone levels below detectible limits (~1.3 ng/g). We did not find correlations between water‐borne corticosterone and plasma corticosterone in any age group (Table 1; Figure 2; ggplot2, Wickham, 2009). Changing units from water‐borne corticosterone pg/SVL/hr to pg/g/hr also produced no correlation with plasma corticosterone (Table 1; Figure 2). In laboratory‐reared salamanders, there was no difference between corticosterone levels for baseline and agitation groups for larvae, metamorphs, or both groups together (*p* > .13; Figure 3). This held true for plasma levels and both units of water‐borne hormones.

**Table 1:** Spearman's correlation of spotted salamander plasma and water‐borne corticosterone levels presented across age groups

Age	Water‐borne unit	*n*	Spearman's *rho*	*p*
Larvae	pg/SVL/hr	16	–0.26	.32
	pg/g/hr	16	–0.33	.21
Metamorphs	pg/SVL/hr	13	–0.24	.42
	pg/g/hr	13	0.12	.69
Adults	pg/SVL/hr	28	–0.03	.88
	pg/g/hr	27	–0.08	.70
Adults (no females)	pg/SVL/hr	26	0.15	.46
	pg/g/hr	26	–0.007	.97
Adults (<3 min blood collection)	pg/SVL/hr	22	–0.11	.64
	pg/g/hr	21	–0.19	.40

Note

Correlations are presented for both water‐borne corticosterone units of measurement: pg/SVL/hr and pg/g/hr. Also included are statistics for adults after removing females and for adults only including individuals whose blood samples were collected within 3 min.

**Figure 4:**
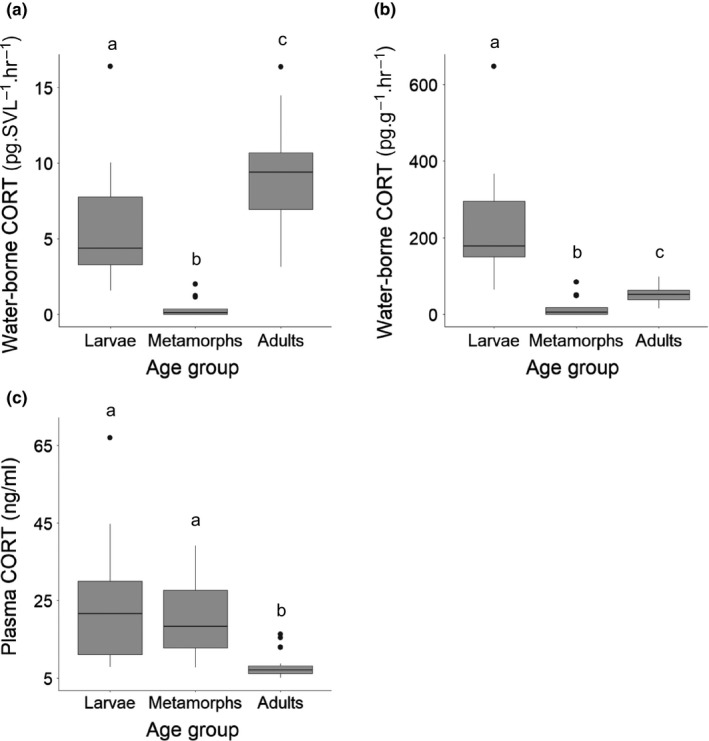
Boxplots displaying the median, interquartile range, lowest and highest observations, and outliers for spotted salamander corticosterone (CORT) levels across age groups (larvae: *n* = 16, metamorphs: *n* = 13, adults: *n* = 28 [27 for water‐borne pg/g/hr]) for water‐borne CORT (a, b) and plasma CORT levels (c). Letters indicate differences in CORT levels between age groups (*p < *.01; Kruskal–Wallis chi‐square; Dunn test)

Water‐borne and plasma corticosterone were different between age groups (larvae, metamorphs and adults; *p* < .01), except when comparing larvae and metamorph plasma levels (*p* = .410; Figure 4). Adults had the highest water‐borne corticosterone in units of pg/SVL/hr; but larvae had the highest levels of water‐borne corticosterone in units of pg/g/hr (Figure 4). Corticosterone was 1.6 × higher in adults than larvae when measured by pg/SVL/hr. However, corticosterone was 4 × lower in adults than larvae when measured by pg/g/hr. This was caused by disparity between mass and SVL. Adult weights were 24 × greater than larvae, whereas SVL was only 3 × greater in adults. Adults had the lowest plasma corticosterone levels (Figure 4).

## 4 DISCUSSION

There was no correlation between corticosterone levels in water‐borne samples and plasma samples for adult spotted salamanders collected in the field or laboratory‐reared larvae and metamorphs. Age group differences in corticosterone were inconsistent between plasma and water‐borne corticosterone and also depended on the method of correcting for body size. Despite the successful chemical validation of assays (including quality controls), we were unable to physiologically validate water‐borne hormones with an association between water‐borne and plasma corticosterone. Our results contrast with those of other amphibian species where positive correlations between plasma levels and water‐borne corticosterone were found in larval western tiger salamanders, adult San Marcos salamanders, common midwife toads, and Túngara frogs (Baugh et al., 2018; Davis et al., 2019; Gabor et al., 2013). There could be two main reasons for why we did not find a correlation. First, water‐borne corticosterone and plasma corticosterone could both be biologically relevant, but inherently differ, inhibiting correlation between the two. Second, our methods might have been insufficient to find a correlation. Below, we discuss our results in more detail.

Chemical validation and quality control are crucial to show the assay is suitable for this species and hormone. Through chemical validation (recovery and parallelism) and quality controls (positive and negative controls), we demonstrated that the assays produced reliable levels of corticosterone for water‐borne and plasma samples (Behringer & Deschner, 2017). Quantitative recovery indicated the assays were able to detect corticosterone following predicted levels. In testing parallelism, the dilution curve was parallel to a standard curve of synthetic corticosterone, showing the water‐borne sample was immunologically similar and could be measured based on the standard curve of the assay (Behringer & Deschner, 2017). Using positive controls, we showed our assays had inter‐ and intra‐assay variation comparable to other published studies of water‐borne hormones (Gabor et al., 2013, 2016). This variation was on the higher end but that was expected as it incorporated variation introduced from the first step of extraction on C18 columns; rather than using pooled samples post‐resuspension, which lacks variation from the extraction process. Finally, we used negative controls to remove any background corticosterone detected in the water separate from what was produced by the salamander.

In addition to chemical validation and quality control, validation of water‐borne sampling requires species‐specific physiological validation. Physiological validation is required to demonstrate that water‐borne corticosterone levels are associated with the physiological state of an animal (Behringer & Deschner, 2017; Madliger et al., 2018). However, we were unable to physiologically validate water‐borne hormones with an association between water‐borne and plasma corticosterone. The first possible explanation for our results is that water‐borne corticosterone and plasma corticosterone may reflect different aspects of the physiological response; such that water‐borne corticosterone cannot be generalized as a reflection of plasma corticosterone. These two sampling methods are producing a unit of measurement unique to that method. It is possible that the unique units of measurement inherently prevent correlations between the two. The two units may also require different interpretations of the data and physiological meaning. Water‐borne hormones are based on the animal's release rate into the water; plasma hormones are a direct measure of corticosterone concentration in the animal's blood. Water‐borne levels might reflect capture and confinement in a novel environment. Additionally, plasma samples reflect a specific point in time, which might be interpreted as reflecting short‐term conditions. Whereas, water‐borne levels measure a rate of release; which might reflect long‐term conditions and represent a more comprehensive measure of corticosterone (Dantzer et al., 2014). Because water‐borne sampling is still relatively new for amphibians, this claim requires additional evidence for species‐specific physiological relevance of the rate.

Despite the lack of correlation between water‐borne and plasma corticosterone; we have evidence that water‐borne corticosterone is biologically meaningful in spotted salamanders. In a previous study, water‐borne corticosterone was associated with salamander body size as well as the water temperature and diameter of vernal pools (Millikin et al., 2019). If water‐borne corticosterone was a random signal, there likely would not have been an association with the body size or the environment. We also detected higher water‐borne corticosterone levels in water that contained salamanders than negative control samples including nanopure water, distilled water, and water enriched with R/O Right Water Conditioner, which is what we used during sampling. We confirmed this with samples sent to the ETC at ONPRC. This indicates that the detected corticosterone is coming from the tested animals.

Another explanation for the association lacking between water‐borne and plasma corticosterone could be methodological. We examined baseline levels after 1 hr of containment, which was adequate to demonstrate correlations between plasma and water‐borne corticosterone in adult common midwife toads and San Marcos salamanders (Gabor et al., 2013). In adult Túngara frogs, plasma and water‐borne corticosterone correlations were found after longer holding periods, 2 hr (Baugh et al., 2018). This might indicate that holding periods longer than 1 hr are required to facilitate a correlation for certain species. Additionally, laboratory effect could have impacted the quantitative values of corticosterone (Fanson, Németh, Ramenofsky, Wingfield, & Buchanan, 2017). By using Spearman's rank correlation, we reduced the impact of variation in the absolute concentration (Fanson et al., 2017). However, it is important to note that for the adult salamander samples we used two different assays and two different laboratories (i.e., ELISA at Duquesne University for water‐borne samples and RIA at the ETC at ONPRC for plasma samples). Using different laboratories may have influenced results for the adults. Despite our demonstrated correlations for ELISA and RIA, using different methods for the adult samples also could have influenced the results because ELISA measured both free corticosterone and conjugated forms (sulfonated corticosterone and glucuronidated corticosterone), while the RIA measured free corticosterone. Laboratory effect is not a concern for the salamander larvae and metamorph data because both water‐borne and plasma samples were processed by RIA at the ETC at ONPRC using methods that measured free corticosterone in both.

Additionally, it is possible that we were unable to find a correlation between water‐borne and plasma corticosterone because corticosterone levels were not sufficiently variable among animals to provide a sufficient range to reveal a correlation. Subtle differences might have not been detected due to variance in the assay. In other studies, animals were injected with ACTH, the secretagogue of corticosterone, to produce a maximal increase in corticosterone. For example, San Marcos salamanders and Túngara frogs produced detectible increases in water‐borne corticosterone levels after ACTH injections (Baugh et al., 2018; Gabor et al., 2016). Along with water‐borne levels, plasma corticosterone was elevated after ACTH injection for Túngara frogs, consistent with the hypothesis that water‐borne corticosterone shows similar patterns to plasma corticosterone (Baugh et al., 2018). We did not incorporate ACTH challenges in our study because the purpose of this validation was to demonstrate water‐borne hormones would reflect plasma levels based on natural variation in physiology, rather than an artificial response. Another strategy to induce a corticosterone response is to handle or agitate the animal. We failed to find an increase in corticosterone due to physical agitation in either the plasma or water‐borne samples, perhaps because chasing the salamander with tweezers was not sufficient to elicit an HPI response. It has been shown that disturbing the containers of salamanders in water increased water‐borne corticosterone levels in Jollyville Plateau salamanders (*Eurycea tonkawae*; Gabor et al., 2016; Gabor et al., 2018), but not in San Marcos salamanders (*E. nana*), and there were conflicting results from different populations of Barton Springs salamanders (*E. sosorum*; Gabor et al., 2016). Our salamanders did display loss of righting reflex, indicating a biological response to our agitation treatment. It is possible that laboratory‐reared metamorphs had elevated baseline corticosterone due to confinement, captivity, or growth and development during metamorphosis, preventing a detectible response (de Assis, Titon, Barsotti, Titon, & Gomes, 2015; Belden, Wingfield, & Kiesecker, 2010; Chambers, Wojdak, Du, & Belden, 2011). It seems these were not influential factors for laboratory‐reared larvae because water‐borne corticosterone levels of laboratory‐reared larvae were similar to those of larvae caught in the field (Millikin et al., 2019). Our field‐caught adults also had baseline plasma corticosterone levels that were similar to other studies (Homan et al., 2003). Additionally, the range of values of water‐borne and plasma corticosterone found in our study was similar to those found in other validation studies that used baseline levels of corticosterone (Gabor et al., 2013) and ACTH injections (Gabor et al., 2016), suggesting that corticosterone was sufficiently variable among individuals to detect a correlation if it existed.

Our results illustrate several additional caveats with sampling water‐borne hormones. The first is the method of correcting for differences in body size. We found different patterns of results depending on how we corrected for body size, SVL versus mass. Related to this, Scott and Ellis (2007) pointed out the difficulty of comparing water‐borne hormones between animals with variation in body size; as we found in the larvae and adults. This is because dividing water‐borne corticosterone by body size can produce artificial differences if the groups differ greatly in body size (Scott & Ellis, 2007). Also, we have evidence that dividing by body size does not completely correct for body size differences even among animals of a similar body size (Millikin et al., 2019). We found after dividing corticosterone pg by total body length, larger animals still had higher corticosterone levels making it necessary to include body size in models to control for its impact when determining predictors of corticosterone levels (Millikin et al., 2019). Another limitation is that perhaps water‐borne hormones should not be compared across different amphibian life stages. Our larvae and metamorphs had similar plasma corticosterone levels. However, metamorphs produced little water‐borne corticosterone. The reabsorption of gills or changes in skin during metamorphosis could alter the passage of corticosterone into the water producing different results than larvae.

Water‐borne hormone sampling has many benefits that make it a method worth further research. Corticosterone detected in plasma is a direct measure of circulating levels in that moment, whereas water‐borne may reflect overall physiological condition (Baugh et al., 2018; Dantzer et al., 2014; Romero & Reed, 2005). Water‐borne sampling is not restricted by animal size and could provide an option for animals too small to sample or detect with other methods. We could not detect whole‐body corticosterone in spotted salamander larvae, making it important to explore whether water‐borne sampling is a viable alternative. Water‐borne sampling could be essential for studying hormone levels in smaller larvae. It also allows for sampling the same individual multiple times and over long periods of time, even of small animals that would otherwise have to be sacrificed (Scott & Ellis, 2007). Many studies have provided evidence that water‐borne corticosterone provides physiologically relevant estimates that are biologically meaningful (Baugh et al., 2018; Charbonnier et al., 2018; Davis et al., 2019; Gabor et al., 2013, 2015, 2016, 2018; Holmes et al., 2016). Because water‐borne hormone sampling is still a new method, additional research is necessary to determine the ideal containment time (1 hr, 2 hr, etc.) for peak corticosterone release in this, and other, species. Future research is necessary to better understand how water‐borne hormone levels vary among individuals and species. It should be determined if water‐borne corticosterone is impacted by amount of handling time, disturbance, or recent interactions with predators. Additionally, the method used to control for body size could influence the relationship between water‐borne and plasma corticosterone. Future research should explore if there are more suitable methods to control for the impact of body size on corticosterone release rate. It is possible that body condition index (MacCracken & Stebbings, 2012) or surface‐area‐to‐volume ratio (Ferreira Amado, Moreno Pinto, & Olalla‐Tárraga, 2019) would more accurately account for body size and control for that influential factor on release rates.

Despite the advantages of water‐borne hormones to assess physiological status, our results illustrate the importance and challenges of physiologically validating the method. There was no correlation between plasma and water‐borne corticosterone levels in spotted salamander larvae or metamorphs in the laboratory, or adults in the field. Water‐borne and plasma corticosterone sampling might provide different data that require separate interpretation methods. Our study indicates water‐borne sampling requires additional research to determine the species‐specific physiological consequences of variation in water‐borne hormone levels. Our study also echoes concerns around the inconsistent pattern of corticosterone levels among life stages and species (Dickens & Romero, 2013; Gormally, Fuller, McVey, & Romero, 2019; Scott & Ellis, 2007). The interpretation of water‐borne hormone levels will depend upon physiological validation and demonstrated biological meaning.

## CONFLICT OF INTEREST

None declared.

## AUTHOR CONTRIBUTIONS

ARM, SKW, and JTA devised the study. JTA and ARM obtained funding. ARM and SKW collected field data. ARM conducted animal care, experiments, and laboratory work with help from technicians and input from SKW and DRD. ARM conducted analysis with advice from SKW and DRD. ITM processed whole‐body samples. ARM wrote the manuscript with significant contributions from all authors.

## Data Availability

Data associated with this study are available in DRYAD https://doi.org/10.5061/dryad.zs7h44j4p
